# Improvements to mood, stress and loneliness following 12-week multivitamin supplementation in older adults: a randomised, placebo-controlled, trial

**DOI:** 10.1038/s41430-024-01517-6

**Published:** 2024-10-03

**Authors:** Sarah Docherty, Mark A. Wetherell, Lynn McInnes, Crystal. F. Haskell-Ramsay

**Affiliations:** Department of Psychology, Northumbria University, Newcastle upon Tyne, UK

**Keywords:** Randomized controlled trials, Nutrition

## Abstract

**Background:**

Research has indicated the potential for multivitamin-mineral (MVM) supplementation to improve aspects of wellbeing and cognitive function in older adults via a range of biological mechanisms. However, outside of cognitive function and mood, this research rarely assesses other outcomes that are pertinent to the daily lives of older adults. The current study aimed to investigate the effectiveness of a MVM supplement on meaningful outcomes of everyday functioning in older adults.

**Methods:**

This randomised, double-blind, placebo-controlled, parallel groups trial investigated the effects of 12-week MVM supplementation on measures of wellbeing, mood, and memory; physical health and activity; and social interaction and loneliness. Outcomes were measured at baseline and after 12 weeks in a sample of 228 (124 female) older adults ( > 70 years).

**Results:**

MVM supplementation had no effect on the primary outcome of wellbeing (*p* = 0.29 in males, *p* = 0.421 in females), but led to increased feelings of friendliness in females (*p* = 0.045). In males, following MVM, there were lower levels of prolonged stress reactivity (*p* = 0.007), lower overall stress reactivity (*p* = 0.019), and lower emotional loneliness (*p* = 0.042).

**Conclusion:**

This study provides novel evidence of increased friendliness and decreased stress reactivity and loneliness following MVM supplementation in older adults. These findings support the exploration of broader functions pertinent to aspects of daily living in older adults. Sex differences in response highlight the importance of exploring effects in men and women separately and support a recommendation for the inclusion of diverse samples in future research that are representative of the population.

## Introduction

Vitamins and minerals are essential for normal cell function and physiological processes, they contribute directly to optimal brin function via a plethora of specific biochemical mechanisms e.g. gene regulation, synthesis of neurotransmitters and hormones and as co-enzymes and precursors of co-factors in enzymatic processes [1]. Supplementation with multivitamins and minerals (MVM) may therefore benefit psychological functioning [2]. Supplementation with MVM has shown a number of benefits to wellbeing across the adult life course, including reductions in stress and anxiety ratings [3–6]. Sex differences in response to MVM have also been reported, with females showing greater improvements in self-reported tiredness and stress [7], which may be due to differing nutritional needs, differences in diet, and varying absorption of micronutrients across sexes [8, 9].

Improvements to cognitive performance have also been shown in healthy young [4] and older adults [10–12] following supplementation of a MVM. However, many studies have failed to find any effects on cognitive function following MVM in older adults [6, 13–15]. Despite a high prevalence of nutritional inadequacies in older adults [16], it has been suggested that null findings may be due to study populations being too well nourished, with greater benefits proposed for older adults with nutritional deficiency [1, 14].

Findings from studies of cognitive function are also limited in terms of their applicability to the real world. It has been argued that traditional laboratory measures of cognition lack ecological validity and do not adequately measure ‘everyday cognition’ for older adults [17]. There has also been little consideration as to whether the outcomes being measured represent meaningful benefits for the research participants. Everyday functioning is a term used to refer to effective functioning in day-to-day life, which is dependent on an individual’s ability to adapt, and is reliant on their sensorimotor, cognitive, personality and social resources [18]. This covers a wide range of domains [19] and is negatively impacted by ageing resulting in increased dependence. There is a clear need for future work to expand on previous work focusing on the effects of MVMs on cognitive function, as this represents just one aspect of successful ageing. Previous nutraceutical research that has focused on a broader range of outcomes linked to everyday functioning is limited and has produced mixed findings when assessing supplementation effects on sleep [20–22]. However, nutritional intervention (alongside functional group-based exercise) was effective at reducing social isolation and improving quality of life in older women with sarcopenia [23].

Research to date suggests that MVM supplementation can positively impact behaviour, but the findings may be limited by the choice of outcomes which lack ecological validity, the propensity to combine data for men and women which may mask some of the effects, and the use of strict inclusion criteria that typically result in healthy, wealthy, well-nourished study populations. The current study aimed to address these limitations by assessing the effects of 12-week MVM supplementation on meaningful outcomes of everyday functioning in adults aged 70 and over, with the primary outcome being subjective wellbeing, as utilised by the Office for National Statistics as part of the Measuring National Wellbeing Programme. To address potential sex differences in response, outcomes were assessed in males and females separately. Finally, the study aimed to be more representative of older adults by widening the inclusion criteria adopted in previous trials.

## Method

### Design

A randomised, placebo-controlled, double-blind, parallel groups design was employed.

### Participants

To be eligible to participate in the study, participants had to be aged 70 or over. Participants were recruited via opportunity sample across the United Kingdom, predominately through social media advertising. Exclusion criteria were minimal, but participants could not take part if they had an allergy to soya. Those who were currently under medical supervision, had epilepsy, a thyroid disorder, haemochromatosis, or suffered from a food allergy were advised to consult their doctor or pharmacist before taking part. Those already taking a MVM supplement, or any individual vitamin/mineral contained in the supplement, had a four-week washout period before taking part, other supplements were considered on a case-by-case basis. A summary of health conditions for the sample is provided in Supplementary Materials.

A priori G*Power analysis calculated based on a medium effect size (*F* = 0.24) seen for mood measures on similar published nutritional interventions [6], indicated that 128 females and 128 males were needed to achieve a power of 0.8. Two-hundred and sixty-six participants (142 female, 124 male) were enroled into the study.

### Materials

#### Treatments

Treatments were randomised by the manufacturer separately for males and females. Treatments were prepared and bottled by the manufacturer in accordance with a computer-generated randomisation list and delivered to the investigational site identified only by their randomisation code. Treatment was consumed as a single tablet, participants were instructed to take one per day with their main meal of the day with water or a cold drink, for 12 weeks. Placebo and MVM were identical in appearance and smell. Blinding was assessed by treatment guess and compliance was measured by participants reporting how many unconsumed tablets were left upon completion of the study. The MVM used was a commercially available product sold by Vitabiotics Ltd, London (Wellwoman 70+ tablets / Wellman 70+ tablets).

Ingredients differed slightly between the male and female MVM, the breakdown of each the treatments are shown in Table 1.

**Table 1:** Breakdown of each ingredient included in the female and male formula, () represents % of daily Nutrient Reference Value, where this is not included there are no recommended daily values.

MVM		
	Female	Male
Lutein Esters	2 mg	2 mg
Co-enzyme Q10	10 mg	10 mg
L-Carnitine	10 mg	20mg
Alpha Lipoic Acid (ALA)	20 mg	20 mg
Phosphatidylcholine	7 mg	7 mg
Silicon	10 mg	10 mg
Citrus Bioflavonoids	10 mg	10 mg
Betacarotene	2 mg	2 mg
Vitamin D (as D3 800 IU)	20 µg (400%)	20 µg (400%)
Vitamin E	12 mg (100%)	12 mg (100%)
Vitamin C	80 mg (100%)	80 mg (100%)
Thiamin (Vitamin B1)	14 mg (1273%)	14 mg (1273%)
Riboflavin (Vitamin B2)	4 mg (286%)	4 mg (286%)
Niacin (Vitamin B3)	30 mg NE (188%)	30 mg NE (188%)
Vitamin B6	10 mg (714%)	10 mg (714%)
Folic Acid	200 µg (100%)	200 µg (100%)
Vitamin B12	100 µg (4000%)	100 µg (4000%)
Biotin	50 µg (100%)	50 µg (100%)
Pantothenic Acid	6 mg (100%)	10 mg (167%)
Iron	10 mg (71%)	6 mg (43%)
Zinc	15 mg (150%)	15 mg (150%)
Copper	1000 µg (100%)	1000 µg (100%)
Manganese	0.5 mg (25%)	0.5 mg (25%)
Selenium	120 µg (218%)	55 µg (100%)
Chromium	75 µg (188%)	75 µg (188%)
Iodine	150 µg (100%)	150 µg (100%)
Siberian Ginseng Extract equiv. to	—	20 mg
Pumpkin Seed Extract equiv. to	—	100 mg
L-Glutathione	—	10 mg
Vitamin A (2666 IU)	—	800 µg RE

#### Questionnaires

A range of measures assessing wellbeing, mood, and memory; physical health and activity; and social interaction and loneliness, were administered online. Please see Table 2 for full descriptions of outcome measures.

**Table 2:** Full description of each outcome measure.

Measure	Description
**Wellbeing, Mood and Memory**	
UK Office of National Statistics (ONS) four subjective well-being questions (ONS4) [47] – Primary Outcome	Consisting of four subjective well-being questions (ONS4) e.g. Overall, how satisfied are you with your life nowadays? Higher scores indicating greater wellbeing.
Perceived Stress Scale (PSS) [48]	The PSS is a 10-item scale which measures the extent to which participants perceive their lives to be overwhelming, uncontrollable and unpredictable. Individual items e.g. “in the last month, how often have you been upset because of something that happened unexpectedly”, are summed to yield a total score. Higher scores indicate greater perceived levels of stress, experienced over the previous month.
The Perceived Stress Reactivity Scale (PSRS) [35]	This 23-item scale measures an individual’s typical stress response to different situations. Items are groups into five subscales (reactivity to social evaluation, reactivity to failure, reactivity to social conflicts, reactivity to work overload, and prolonged reactivity) and one overall score of total stress reactivity. Higher scores indicate higher stress response.
The Hospital Anxiety and Depression Scale [49]	This is a 14-item scale (7 questions relating to anxiety e.g. I feel tense or ‘wound up’ and 7 to depression e.g. I feel cheerful). Higher scores indicate higher levels.
The Profile of Mood States [50]	This comprises 65 adjectives (e.g., helpful, unhappy), participants are asked to rate how much they have felt this in the last week. Scores are summed to give global scores for seven subscales: tension, depression, anger, fatigue, confusion, vigour, and friendliness.
The Prospective and Retrospective Memory Questionnaire (PRMQ) [51]	This is a 16-item scale that measures everyday memory failures e.g. Do you forget something that you were told a few minutes before. Higher scores indicate more everyday memory failures.
**Physical Health and Activity**	
The Cohen–Hoberman Inventory of Physical Symptoms (CHIPS) [52]	A measure of perceived burden as a result of physical symptoms. This consists of 33 common symptoms (e.g., ‘back pain’, ‘constipation’ and ‘heart pounding or racing’) experienced over the previous two weeks. Higher scores indicating greater perceived burden due to physical symptoms and poorer health.
The Medical Outcomes Study short form questionnaire (SF-20) [53]	Measuring general health across six domains: physical functioning, role functioning, social functioning, mental health, health perceptions, and pain. Higher scores representing better outcomes, except for pain in which lower scores represent lower levels.
The Pittsburgh Sleep Quality Inventory (PSQI) [54]	This measures sleep quality and patterns over seven domains e.g. sleep disturbances, which are then used to create a global sleep score, higher scores indicating poorer sleep quality.
The Yale Physical Activity Survey [55]	A questionnaire developed to assess physical activity in older adults. Consisting of comprehensive physical work, exercise, and recreational activities checklist to assess time spent in these types of activities during a typical week in the past month. Higher scores indicating greater physical activity.
Falls Efficacy Scale International [56]	A 16-item questionnaire which states different activities (e.g., ‘walking on a slippery surface’) and participants are asked to rate how much they would be concerned with falling while doing this activity. Higher scores represent greater concern with falling.
**Social Interaction and Loneliness**	
The Convoy Method [57]	This measures social network size. Participants must write out people in their social network, and rate the quality of their relationship. Higher scores indicate greater social networks and better relationship quality.
The Lubben Social Network Scale [58]	This is made up of 12 questions (6 about family ties and 6 friendship ties) relating to different aspects of social networks such active social network, perceived support network and perceived confidant network. Higher scores representing a larger social network.
De Jong Gierveld Loneliness Scale [59]	An 11-item scale which gives indices of emotional, social and total loneliness. Participants are presented with a list of statements (six relating to emotional loneliness and five social loneliness) and must indicate the extent to which the statement applies to their situation. Higher scores indicate greater loneliness.
**Nutritional Intake**	
Food Frequency Questionnaire (FFQ) [60]	This FFQ was developed during the European Prospective Investigation into Cancer Study (EPIC-Norfolk), which has become widely used as a measure of nutritional intake. The questionnaire lists 130 food products and participants are asked to indicate the frequency of consumption.

### Procedure

The original preregistered protocol of this study was developed as a face-to-face laboratory study. However, due to the COVID-19 pandemic, the protocol had to be adapted to run as an online intervention, procedure outlined here. For original face-to-face protocol see Clinical Trials.gov (reference: NCT04112732).

Participants were required to complete a telephone screening session which comprised a briefing on the requirements of the study, signing of a virtual consent form, and provision of demographic information (including date of birth, medical conditions, medication, daily alcohol and caffeine consumption, and approximation of height and weight). Participants were then sent treatment and a treatment diary via post. When this was received, participants were required to complete testing session 1 as an online appointment. Prior to the morning of testing session 1, participants were asked to avoid alcohol for 24 h and caffeine for 12 h, and to eat a standardised breakfast of cereal and/or toast. This was to ensure standardisation across sessions and participants, and to minimise the impact of other factors, such as the acute psychoactive effects of caffeine which may impact mood [24]. A link to the online questionnaire was sent via email, adherence to protocol was assessed via the questionnaire, and then participants could complete the questionnaires at their own pace. When this was completed, participants were asked to start consuming the tablets they had been sent, one tablet per day with their main meal of the day with water or a cold drink, and to record the time of this daily in the diary provided, along with any adverse events and concomitant medications taken throughout the trial. After 12 weeks (± 5 days) participants completed another online session. In addition to the tools completed at session 1 participants were also asked to record any changes in medication, adverse events, the number of remaining tablets, to complete the treatment guess questionnaire, and were presented a full debrief sheet when the questionnaires were completed.

### Statistical analysis

All data was analysed in IBM SPSS statistics (v 27.0). Outcome data were cleaned by generating box plots for each outcome variable to identify potential outliers.

Data was analysed separately for males and females using one-way independent groups ANCOVAs. An overall score was calculated for EPIC FFQ data as a measure of diet quality. Quartiles were calculated for each nutrient of interest (those included in the MVM product), these were then counted and participants were categorised as having a high or low diet quality based on the most frequent quartile. Treatment was an independent factor with two levels: MVM and placebo. Diet quality was also included in the model as an independent factor, but removed if no significant interaction with treatment was observed. The dependent measures were the relevant outcomes for each questionnaire at the final session (after 12 weeks intervention). The relevant outcome for each questionnaire at baseline (before supplementation) was included as a covariate. Both primary and secondary outcomes were analysed following the same procedure. Due to the volume of secondary outcomes, results should be interpreted with caution. All participants who provided post dose assessment data were included in the Intent-To-Treat population.

Before ANCOVA could be run, homogeneity of regression slopes assumption was checked for each outcome. ANCOVA is still robust when the assumption of equal regression slopes is violated if group sizes are equal [25]. As group sizes were equal ANCOVAs were run and reported as planned.

Prior to primary analysis, demographic and dietary differences across treatment groups and between sexes were assessed with one-way ANOVAs.

Treatment guess was analysed by *χ* ^2^ test.

## Results

Of the 266 participants enroled, 228 participants provided post-dose assessment data (124 female, 104 male). In both males and females, there were no significant differences between treatment groups for age, years spent in education, daily alcohol or caffeine intake, and BMI. In men, daily intake of fruit was significantly lower in the MVM group at baseline [*F* (1,85) = 5.55, *p* = 0.021], dietary intake of manganese was also significantly lower in the MVM group at baseline [*F* (1, 85) = 5.16, *p* = 0.026]. Men also consumed significantly more alcohol than women [*F* (1226) = 6.18, *p* = 0.014], and significantly less fruit [*F* (1196) = 5.74, *p* = 0.018] and vegetables [*F* (1196) = 7.78, *p* = 0.006].

**Fig. 1:**
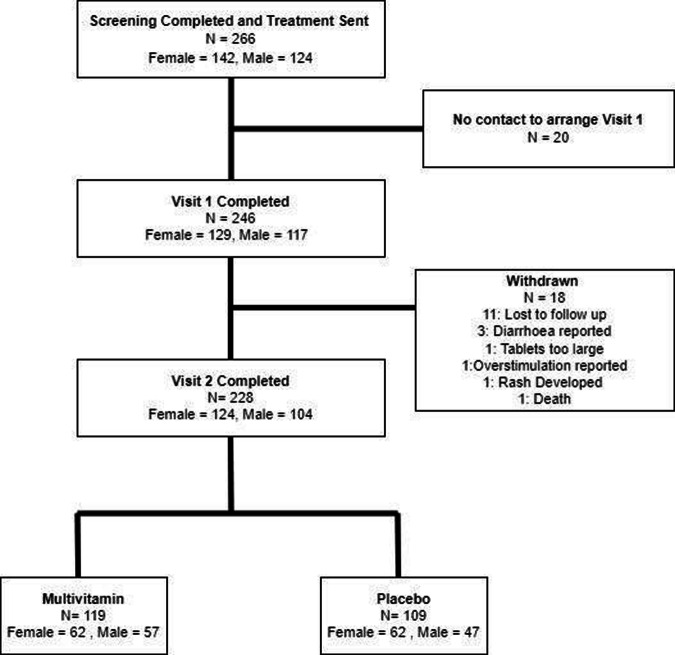
Final participation disposition throughout the trial, cumulating in the 228 participants who completed the study *N* = number of participants.

Participant disposition is displayed in Fig. 1 and demographic data in Table 3.

**Table 3:** Participant demographic information for the 228 subjects who completed the study (124 females, 104 males).

	Female				Male				
	Multivitamin	Placebo	Total	*p*	Multivitamin	Placebo	Total	*p*	*p (between sex)*
Age (years)	73.50 (3.63)	74.19 (4.35)	73.85 (4.00)	0.377	74.35 (3.55)	74.47 (3.97)	74.40 (3.73)	0.874	0.281
BMI (kg/m^2^)	26.54 (5.80)	26.65 (4.04)	26.59 (4.98)	0.909	27.96 (4.87)	28.02 (5.41)	27.99 (5.10)	0.954	0.038
Years in Education	14.86 (3.29)	15.43 (2.66)	15.15 (2.99)	0.295	15.40 (2.70)	15.79 (2.60)	15.58 (2.65)	0.465	0.254
Caffeine (mg per day)	245.30 (145.82)	255.26 (143.08)	250.28 (143.95)	0.702	239.71 (144.80)	236.24 (135.54)	238.14 (140.03)	0.901	0.521
Alcohol (units per day)	0.70 (0.96)	0.71 (0.83)	0.71 (0.90)	0.973	1.05 (1.22)	1.00 (0.83)	1.03 (1.06)	0.818	0.007
Fruit (grams per day)	279.34 (168.74)	320.34 (314.48)	299.29 (250.14)	0.391	191.14 (130.10)	264.66 (160.31)	226.63 (149.26)	0.021	0.018
Vegetables (grams per day)	322.21 (128.15)	367.58 (228.04)	344.28 (184.22)	0.196	256.20 (129.75)	294.52 (187.40)	274.70 (160.40)	0.268	0.006

Means, Std. Deviation (SD), split by sex and treatment report. *p* value reported for comparison between treatment groups within each sex, final column denotes differences between females and males (totals) for each demographic variable reported.

There were no interactions between treatment and diet quality, therefore diet quality was not included as a factor in the final analysis. See Table 4 for nutrient values in the high and low diet quality groups.

**Table 4:** Mean nutrient intake for each vitamin/mineral for the low and high intake group, split by treatment and sex. RDA shows recommended daily allowance for each sex.

	Multivitamin		Placebo		
	Low	High	Low	High	RDA
**Beta_carotene**					
Male	2548.66	3401.48	2322.45	3994.70	—
Female	3138.07	4177.39	2697.17	5110.20	—
**Copper (mg)**					
Male	0.91	1.35	0.96	1.62	1.2
Female	0.96	1.56	0.87	1.52	1.2
**Iron (mg)**					
Male	8.99	12.82	8.71	14.81	8.7
Female	8.91	14.09	9.12	14.29	8.7
**Folate (µg)**					
Male	239.06	332.83	235.64	382.07	200
Female	251.09	368.65	246.28	409.77	200
**Iodine (µg)**					
Male	117.61	176.86	118.23	176.51	140
Female	128.27	181.28	129.27	175.08	140
**Manganese (mg)**					
Male	2.87	3.80	2.92	5.25	2.3
Female	3.02	4.53	2.82	5.13	1.8
**Niacin (mg)**					
Male	17.73	25.20	15.93	29.05	15.5
Female	17.20	28.18	17.80	27.76	12.6
**Vitamin A (retinol) (µg)**					
Male	988.24	1297.43	907.06	1955.32	700
Female	1106.72	1533.10	840.58	1675.43	600
**Vitamin B2 (µg)**					
Male	1.58	2.25	1.50	2.41	1.3
Female	1.66	2.31	1.57	2.39	1.1
**Selenium (µg)**					75
Male	48.16	70.44	46.70	80.16	60
Female	47.96	77.67	50.70	76.52	
**Vitamin B1 (mg)**					
Male	1.17	1.64	1.15	1.95	0.9
Female	1.14	1.87	1.19	1.91	0.8
**Vitamin B12 (µg)**					
Male	5.17	7.83	4.57	7.34	1.5
Female	5.11	8.17	4.88	7.94	1.5
**Vitamin B6 (mg)**					
Male	1.70	2.48	1.78	2.75	1.4
Female	1.73	2.75	1.75	2.70	1.2
**Vitamin C (mg)**					
Male	92.51	115.85	100.11	140.42	40
Female	101.44	156.75	106.15	175.17	40
**Vitamin D (µg)**					
Male	2.05	3.65	1.91	3.29	10
Female	2.25	3.61	2.36	3.68	10
**Vitamin E (mg)**					
Male	9.83	14.60	9.03	15.34	15
Female	10.46	14.19	9.93	16.51	15
**Zinc (mg)**					
Male	7.03	9.79	7.13	11.91	9.5
Female	7.07	10.90	6.93	11.41	7.0

**Fig. 2:**
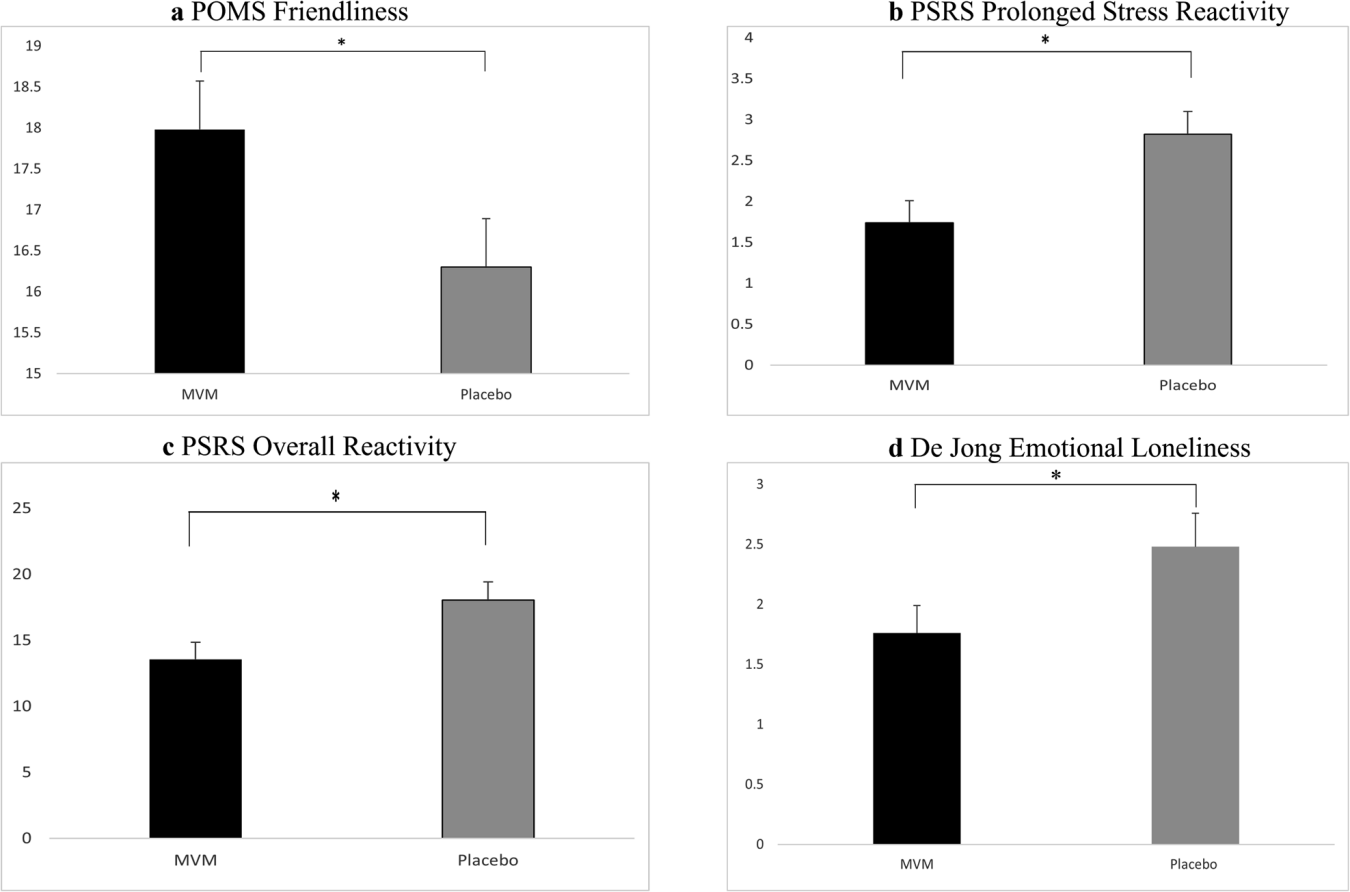
Significant treatment effects. Adjusted means and standard error for treatment effects on **a** POMS friendliness score in females, **b** PSRS Prolonged Stress Reactivity in males, **c** PSRS Overall Reactivity in males and **d** De Jong emotional loneliness score in males. *p < 0.05.

In females, there was a significant effect on POMS friendliness [*F* (1118) = 4.10, *p* = 0.045, ^h^p^2^ = 0.034], with those who received MVM (mean = 17.98. SE = 0.58) reporting higher levels of friendliness compared to placebo (mean = 16.30, SE = 0.59). See Fig. 2a.

There was a significant effect of treatment in males on prolonged stress reactivity [*F* (183) = 7.78, *p* = 0.007, h_p_^2^ = 0.086], those who received MVM reported lower levels of prolonged reactivity (mean = 1.74, SE = 0.27) compared to those who received placebo (mean = 2.82 SE = 0.28). This effect was also found on overall perceived stress reactivity in males [*F* (1, 83) = 5.72, *p* = 0.019, h_p_^2^ = 0.064], with lower levels following MVM (mean = 13.56, SE = 1.29) compared to placebo (mean = 18.05, SE = 1.36). See Fig. 2b, c.

In males, there was a significant effect of treatment on De Jong-Gierveld emotional loneliness scale [*F* (1101) = 4.23, *p* =0.042, h ^2^ = 0.040], with those having MVM reporting significantly lower levels of emotional loneliness (mean = 1.76, SE = 0.23) compared to placebo (mean = 2.48, SE = 0.28). See Fig. 2d.

Compliance (assessed via number of tablets reported to be left upon completion) was observed to be very good on average in both females (100.3% MVM, 100.4% Placebo) and males (99.5% MVM, 99.4% Placebo). One-way ANOVA showed no significant difference for compliance percentage by treatment group for females [*F* (1122) = 0.007, *p* = 0.935] or males [*F* (1102) = 0.002, *p* = 0.968].

In females, 68% of participants in the placebo group and 68% in the MVM group believed they had received placebo. In males, 82% in the placebo group and 82% in the MVM group believed they had consumed placebo. There was no significant difference in participants’ ability to identify whether they had consumed multivitamin or placebo in females [χ2 (1) = 0.004, *p* = 0.951] or males [χ2 (1) = 0.003, *p* = 0.958].

For full data see Table 5a–c.

**Table 5:** **a** Unadjusted Mean (SD), *F* values and *P* values for all wellbeing, mood and memory outcomes, split by Sex and Treatment.

	Males					Females		
	Multivitamin	Placebo	*F* Value	*P* Value	Multivitamin	Placebo	*F* Value	*P* Value
Office National Statistics Wellbeing	30.53 (5.82)	30.98 (5.48)	0.29	0.590	29.35 (5.82)	29.97 (6.63)	0.65	0.421
Perceived Stress Scale	10.59 (5.50)	12.77 (7.54)	1.69	0.196	12.15 (6.31)	13.48 (6.20)	1.52	0.220
***Hospital Anxiety and Depression*** ***Scale***								
Anxiety	4.14 (3.59)	4.83 (4.42)	0.009	0.927	5.02 (3.89)	4.73 (3.14)	0.02	0.891
Depression	3.11 (2.93)	3.98 (3.49)	0.66	0.418	3.97 (3.34)	3.69 (3.05)	0.11	0.738
***Profile of Mood States***								
Tension – Anxiety	4.31 (6.49)	6.18 (7.90)	0.82	0.368	5.00 (6.86)	5.52 (6.00)	0.24	0.626
Depression – Dejection	4.04 (8.03)	5.76 (8.84)	0.35	0.558	4.64 (8.29)	5.43 (8.16)	0.45	0.506
Anger – Hostility	2.80 (3.82)	3.89 (6.72)	0.16	0.687	2.62 (4.89)	3.45 (5.13)	1.36	0.247
Vigour – Activity	21.33 (7.56)	18.47 (6.84)	2.40	0.124	18.62 (7.40)	18.93 (8.73)	0.05	0.822
Fatigue – Inertia	3.56 (5.65)	4.98 (6.50)	0.85	0.359	4.00 (6.09)	5.17 (6.43)	1.17	0.282
Confusion- Bewilderment	4.56 (4.98)	6.56 (5.76)	1.86	0.176	6.52 (5.75)	6.75 (5.40)	0.20	0.653
Friendliness	17.75 (3.70)	16.27 (3.32)	3.60	0.061	17.95 (4.17)	16.33 (5.04)	4.10	0.045
Total Mood Disturbance	-2.05 (29.85)	8.89 (37.73)	1.20	0.277	4.16 (32.70)	7.38 (33.90)	0.41	0.521
**Perceived Stress Reactivity Scale**								
Prolonged Reactivity	1.67 (1.91)	2.90 (1.95)	7.78	0.007	2.31 (1.65)	2.50 (1.72)	0.29	0.592
Work Overload	2.29 (2.53)	3.34 (2.63)	2.30	0.133	2.55 (2.41)	2.62 (2.55)	0.01	0.905
Social Conflict	3.69 (2.11)	4.71 (2.53)	3.63	0.060	4.53 (2.32)	4.60 (2.60)	0.02	0.894
Failure	3.16 (1.62)	3.76 (1.59)	3.04	0.085	3.14 (1.65)	3.34 (1.64)	0.34	0.562
Social Evaluation	2.60 (2.29)	3.51 (2.55)	3.45	0.067	2.96 (2.30)	2.76 (2.30)	0.12	0.726
Overall	13.40 (8.28)	18.22 (9.28)	5.72	0.019	15.49 (8.53)	15.82 (8.81)	0.05	0.832
**Prospective and Retrospective** **Memory Questionnaire**								
Prospective Memory	18.91 (7.54)	20.10 (5.59)	0.18	0.676	20.02 (6.78)	19.82 (6.66)	0.00	0.999
Retrospective Memory	17.54 (7.78)	17.96 (5.86)	0.02	0.890	18.98 (7.44)	18.42 (7.09)	0.11	0.737
**b**.								
Cohen Hoberman Inventory of Physical Symptoms	10.42 (12.08)	12.81 (12.58)	0.00	0.996	12.92 (14.36)	15.32 (12.90)	1.57	0.213
Pittsburgh Sleep Quality Index	6.55 (2.87)	6.21 (3.35)	0.40	0.530	7.16 (3.97)	6.44 (3.58)	0.91	0.343
Instrumental Activities of Daily Living	7.60 (0.89)	7.64 (0.75)	0.01	0.924	7.57 (0.93)	7.67 (0.77)	0.32	0.575
Falling Efficacy Scale-International	21.58 (9.95)	20.85 (6.22)	0.42	0.520	24.47 (10.15)	24.57 (9.69)	0.00	0.949
***Yale Physical Activity Scale***								
Total Time	32.19 (19.47)	27.54 (15.56)	1.20	0.276	31.34 (21.37)	32.86 (20.41)	0.13	0.721
Energy Expenditure	124.51 (70.76)	103.60 (62.63)	2.37	0.127	110.02 (76.93)	116.79 (72.81)	0.31	0.578
Activity Dimension	56.05 (30.94)	51.81 (23.17)	0.37	0.545	47.66 (26.25)	52.73 (30.58)	1.37	0.245
***SF-20***								
Physical Function	69.73 (32.03)	78.71 (19.49)	2.80	0.097	66.13 (29.98)	64.91 (30.26)	0.04	0.850
Role Functioning	82.02 (38.31)	87.77 (26.00)	0.59	0.433	78.63 (38.64)	80.24 (36.53)	0.30	0.588
Social Functioning	89.82 (25.95)	95.32 (13.96)	1.95	0.165	88.38 (29.10)	87.10 (25.38)	0.00	0.948
Mental Health	83.19 (15.58)	79.23 (19.21)	0.50	0.480	78.65 (17.35)	75.42 (19.32)	1.08	0.301
Health Perceptions	71.09 (28.36)	74.41 (18.04)	0.42	0.520	72.36 (25.14)	68.00 (27.71)	0.80	0.374
Pain	36.14 (26.84)	39.57 (27.18)	0.40	0.531	39.35 (27.81)	38.06 (25.79)	0.07	0.796
**c**.								
Lubben Social Network Scale	31.16 (10.13)	29.95 (8.86)	0.86	0.356	32.19 (9.18)	33.74 (8.40)	0.97	0.327
***De Jong-Gierveld Loneliness Scale***								
Social Loneliness	2.02 (1.81)	2.49 (1.88)	0.80	0.373	1.87 (1.82)	1.66 (1.81)	0.27	0.602
Emotional Loneliness	1.68 (1.70)	2.57 (2.00)	4.23	0.042	2.11 (1.85)	2.07 (1.96)	0.03	0.873
Total Loneliness	3.70 (2.49)	5.06 (3.55)	3.33	0.071	3.98 (3.35)	3.72 (3.41)	0.14	0.714
***Convoy Model Social Relations***								
Inner Circle Relationships	4.83 (3.13)	4.78 (2.80)	0.00	0.957	6.67 (3.87)	6.05 (4.37)	0.63	0.430
Inner Relationship Quality	8.40 (2.30)	8.53 (1.74)	0.01	0.924	8.83 (1.56)	8.38 (2.20)	1.60	0.208
Middle Circle Relationships	3.43 (2.65)	3.20 (2.26)	0.00	0.991	4.64 (4.61)	4.36 (3.42)	0.26	0.613
Middle Relationship Quality	6.64 (2.27)	6.54 (2.63)	0.01	0.937	6.62 (2.42)	6.49 (2.26)	0.09	0.763
Outer Circle Relationships	3.11 (3.42)	2.43 (2.35)	1.17	0.283	4.07 (4.16)	3.21 (2.68)	1.13	0.290
Outer Relationship Quality	5.42 (3.12)	5.15 (3.38)	0.17	0.680	5.54 (3.26)	5.49 (3.08)	0.01	0.938
Total Relationships	11.37 (7.34)	10.41 (5.38)	0.16	0.687	15.38 (10.73)	13.62 (8.44)	0.61	0.437
Total Relationship Quality	7.31 (1.88)	7.52 (1.56)	0.25	0.617	7.71 (1.59)	7.51 (1.35)	0.71	0.400

**b** Unadjusted Mean (SD), *F* values and *P* Values for all physical health and activity outcomes, split by Sex and Treatment.

**c** Unadjusted Mean (SD), *F* values and *P* Values for all social interaction and loneliness outcomes, split by Sex and Treatment.

## Discussion

This trial explored the effects of 12 weeks’ supplementation with a multivitamin-mineral (MVM) on outcomes of everyday functioning in ‘everyday’ adults aged 70 and over. MVM resulted in a significant improvement in the mood state of friendliness in females and significantly reduced prolonged stress reactivity, overall perceived stress reactivity, and emotional loneliness in males. However, there was no effect on the primary outcome of wellbeing.

Firstly, it must be acknowledged that many of the outcome measures, including the primary outcome, showed no improvement following MVM supplementation. This may be due to issues with MVM absorption and bioavailability [26], particularly in older adults [27]. Inclusion of biomarkers of vitamin and mineral status pre and post supplementation was not possible in the current study due to the global pandemic but is essential in future studies to establish whether MVMs are having the theorised effect on nutritional status. Furthermore, although acute effects of MVMs have been observed, longer than 12 weeks’ supplementation may be needed time to see any improvements in certain outcomes, particularly self-reported health, and physical activity. It has been suggested that randomised controlled trials spanning longer than 12 months are needed to determine the true efficacy of MVM supplementation [28, 29].

The finding of improved friendliness extends previous work showing improvements to subjective feelings of mood [30–32], and specific improvement to mood in females [31]. A greater sensitivity to mood improvements following supplementation may be due to the known higher risk for depression in females [33]. This is supported by the lower wellbeing scores reported at baseline in the current study, suggesting that females have greater capacity to improve facets of their wellbeing.

The reduction in prolonged and overall perceived stress reactivity in males, supports previous work showing lower perceived stress in healthy males after 28 [3, 5] and 33 days’ supplementation [4] and in older adult females following acute consumption of a MVM (3 h post dose) [6]. It is, however, important to note that the reductions in the current study were in perceived stress reactivity, which is a different construct to perceived stress assessed in previous studies. Overall perceived stress reactivity refers to subjective reaction to different stressful situations, specifically focusing on person-environmental interactions, which makes this construct more sensitive to change [34]. Furthermore, prolonged stress reactivity refers to the difficulty relaxing after high workload [35]. Stress reactivity is particularly important to older adults as this has been shown to be related to future health and disease outcomes, with higher reactivity linked to poorer health [36]. No previous MVM trials have assessed stress reactivity, so these results add important and novel evidence to the field. That these greater reductions were observed in males only, is unexpected given that females usually report higher levels of stress [37], (including in the current study) and that sex differences in stress response to MVM have previously favoured females [7]. The absence of stress reduction in females may be indicative of greater nutrient deficiencies in male participants at baseline. This suggestion is supported by males, in the current study, reporting drinking more alcohol and eating less fruit and vegetables than females. Although biomarkers of nutrition status are needed to draw firm conclusions on the mechanism, the findings provide further support for the need to analyse effects of nutrition separately in males and females.

Perhaps the most surprising finding was that MVM supplementation reduced emotional loneliness in males. This facet of social interaction has not been previously assessed, in relation to supplementation, probably because of the lack of an obvious biological mechanism. However, emerging research has shown that the origin of loneliness may have a biological underpinning related to stress [38], with stress having physiological affects which can lead to worsened physical health, and reduced ability to socialise. This can be amplified in older adults who may already be experiencing deteriorating health. Given than those aged over 65 have the highest levels of loneliness in the UK [39], avenues to combat this are warranted. As evidenced in the current study, MVM supplementation can affect feelings of stress in older adult populations. If supplementation can reduce feelings of stress, this may reduce one pathway to loneliness in older adults, but early intervention may be key. Subjective feelings of loneliness have also been associated with risk of malnutrition [40, 41], therefore supplementation in older adults who may struggle to consume recommended levels of micronutrients via their diet could be suggested. This is a novel, but promising area of research which warrants further exploration, especially given the impact of loneliness on health and wellbeing [42].

Finally, although the study had minimal exclusion criteria, the participants included were still relatively healthy. The average BMI and alcohol intake were below the national averages based on sex and age [43, 44], and participants were well educated which is linked to a better diet quality [45]. Referring to the dietary intake in the current sample (outlined in Table 4), it is evident that participants were consuming above the recommended daily allowance for all vitamins and minerals of interest (except for copper, iodine, selenium and vitamin D), even in the low intake group. This may explain the lack of interactions between diet quality and MVM in the current study as participants were well nourished at baseline. For many vitamins and minerals this was also above the RDA for younger adults. Despite attempting to widen participation by having minimal exclusion due to self-selection bias this has not been achieved adequately. It is possible that effects on a wider range of measures would be observed in older adults with clear deficiencies. Going forward, it is imperative that researchers target specific participants in future research to overcome the self-selection bias shown in nutritional trials [46], with prior dietary screening before enrolment recommended.

To conclude, 12 weeks of MVM supplementation in adults aged 70 and over was shown to improve feelings of friendliness in females, and to reduce perceived stress reactivity and emotional loneliness in males. These novel findings of effects on friendliness, stress reactivity and loneliness add to the literature demonstrating improvements following supplementation. Based on the results of this study it is suggested future work should encompass a wider range of outcome measures, moving away from the traditional cognitive testing models, consider sex differences in response to supplementation, and target specific demographics who will benefit the greatest from supplementation.

## Supplementary information

Table S1

## Data Availability

Data available upon request.

## References

[1] Kennedy DO, Haskell CF. Vitamins and cognition: what is the evidence? Drugs. 2011;71:1957–71. 21985165 10.2165/11594130-000000000-00000

[2] Kennedy DO, Stevenson EJ, Jackson PA, Dunn S, Wishart K, Bieri G, et al. Multivitamins and minerals modulate whole-body energy metabolism and cerebral blood-flow during cognitive task performance: a double-blind, randomised, placebo-controlled trial. Nutr Metab. 2016;13:1–16. 10.1186/s12986-016-0071-4 PMC4750202 26870152

[3] Carroll D, Ring C, Suter M, Willemsen G. The effects of an oral multivitamin combination with calcium, magnesium, and zinc on psychological well-being in healthy young male volunteers: a double-blind placebo-controlled trial. Psychopharmacology. 2000;150:220–5. 10907676 10.1007/s002130000406

[4] Kennedy DO, Veasey R, Watson A, Dodd F, Jones E, Maggini S, et al. Effects of high-dose B vitamin complex with vitamin C and minerals on subjective mood and performance in healthy males. Psychopharmacology. 2010;211:55–68. 20454891 10.1007/s00213-010-1870-3 PMC2885294

[5] Harris E, Kirk J, Rowsell R, Vitetta L, Sali A, Scholey AB, et al. The effect of multivitamin supplementation on mood and stress in healthy older men. Hum Psychopharmacol. 2011;26:560–7. 22095836 10.1002/hup.1245

[6] Macpherson H, Rowsell R, Cox K, Scholey A, Pipingas A. Acute mood but not cognitive improvements following administration of a single multivitamin and mineral supplement in healthy women aged 50 and above: a randomised controlled trial. Age. 2015;37:1–10. 25903286 10.1007/s11357-015-9782-0 PMC4408300

[7] Dodd F, Kennedy D, Stevenson E, Veasey R, Walker K, Reed S, et al. Acute and chronic effects of multivitamin/mineral supplementation on objective and subjective energy measures. Nutr Metab. 2020;17:1–14. 10.1186/s12986-020-00435-1 PMC7038616 32123534

[8] Prentice A. Sex differences in requirements for micronutrients across the lifecourse. Proc Nutr Soc. 2021;80:356–64. 33663641 10.1017/S0029665121000550 PMC7613588

[9] Bennett E, Peters SA, Woodward M. Sex differences in macronutrient intake and adherence to dietary recommendations: findings from the UK Biobank. BMJ Open. 2018;8:e020017. 29691247 10.1136/bmjopen-2017-020017 PMC5922487

[10] Macpherson H, Ellis KA, Sali A, Pipingas A. Memory improvements in elderly women following 16 weeks treatment with a combined multivitamin, mineral and herbal supplement: a randomized controlled trial. Psychopharmacology. 2012;220:351–65. 22006207 10.1007/s00213-011-2481-3

[11] Harris E, Macpherson H, Vitetta L, Kirk J, Sali A, Pipingas A. Effects of a multivitamin, mineral and herbal supplement on cognition and blood biomarkers in older men: a randomised, placebo‐controlled trial. Hum Psychopharmacol. 2012;27:370–7. 22711385 10.1002/hup.2236

[12] Yeung LK, Alschuler DM, Wall M, Luttmann-Gibson H, Copeland T, Hale C, et al. Multivitamin supplementation improves memory in older adults: a randomized clinical trial. Am J Clin Nutr. 2023;118:273–282. 37244291 10.1016/j.ajcnut.2023.05.011 PMC10375458

[13] Grodstein F, O’Brien J, Kang JH, Dushkes R, Cook NR, Okereke O, et al. Long-term multivitamin supplementation and cognitive function in men: a randomized trial. Ann Intern Med. 2013;159:806–14. 24490265 10.7326/0003-4819-159-12-201312170-00006 PMC3858850

[14] Harris E, Macpherson H, Pipingas A. Improved blood biomarkers but no cognitive effects from 16 weeks of multivitamin supplementation in healthy older adults. Nutrients. 2015;7:3796–812. 25996285 10.3390/nu7053796 PMC4446780

[15] McNeill G, Avenell A, Campbell MK, Cook JA, Hannaford PC, Kilonzo MM, et al. Effect of multivitamin and multimineral supplementation on cognitive function in men and women aged 65 years and over: a randomised controlled trial. Nutr J. 2007;6:1–5. 17474991 10.1186/1475-2891-6-10 PMC1872030

[16] De Groot C, Van Den Broek T, Van Staveren W. Energy intake and micronutrient intake in elderly Europeans: seeking the minimum requirement in the SENECA study. Age Ageing. 1999;28:469–74. 10529042 10.1093/ageing/28.5.469

[17] Bielak AA, Hatt CR, Diehl M. Cognitive performance in adults’ daily lives: Is there a lab-life gap? Res Hum Dev. 2017;14:219–33. 37771386 10.1080/15427609.2017.1340050 PMC10538579

[18] Baltes MM, Lang FR. Everyday functioning and successful aging: the impact of resources. Psychol Aging. 1997;12:433. 9308091 10.1037//0882-7974.12.3.433

[19] Farias ST, Mungas D, Reed BR, Harvey D, Cahn-Weiner D, DeCarli C. MCI is associated with deficits in everyday functioning. Alzheimer Dis Assoc Disord. 2006;20:217. 17132965 10.1097/01.wad.0000213849.51495.d9 PMC2880610

[20] Wouters-Wesseling W, Van Hooijdonk C, Wagenaar L, Bindels J, De Groot L, Van Staveren W. The effect of a liquid nutrition supplement on body composition and physical functioning in elderly people. Clin Nutr. 2003;22:371–7. 12880604 10.1016/s0261-5614(03)00034-7

[21] Sarris J, Cox KH, Camfield DA, Scholey A, Stough C, Fogg E, et al. Participant experiences from chronic administration of a multivitamin versus placebo on subjective health and wellbeing: a double-blind qualitative analysis of a randomised controlled trial. Nutr J. 2012;11:1–10. 23241329 10.1186/1475-2891-11-110 PMC3545984

[22] Lichstein KL, Payne KL, Soeffing JP, Durrence HH, Taylor DJ, Riedel BW, et al. Vitamins and sleep: an exploratory study. Sleep Med. 2007;9:27–32. 17825610 10.1016/j.sleep.2006.12.009 PMC2174691

[23] Pinheiro HA, Cerceau VR, Pereira LC, Funghetto SS, Menezes RLd. Nutritional intervention and functional exercises improve depression, loneliness and quality of life in elderly women with sarcopenia: a randomized clinical trial. Fisioterapia em Movimento. 2020;33:e003332.

[24] Liguori A, Hughes JR, Grass JA. Absorption and subjective effects of caffeine from coffee, cola and capsules. Pharmacol Biochem Behav. 1997;58:721–6. 9329065 10.1016/s0091-3057(97)00003-8

[25] Levy KJ. A Monte Carlo study of analysis of covariance under violations of the assumptions of normality and equal regression slopes. Educ Psychol Meas. 1980;40:835–40.

[26] Yetley EA. Multivitamin and multimineral dietary supplements: definitions, characterization, bioavailability, and drug interactions. Am J Clin Nutr. 2007;85:269S–76S. 17209208 10.1093/ajcn/85.1.269S

[27] Han S. The impact of age on acute bioavailability of minerals from a multivitamin and mineral supplement: ResearchSpace@ Auckland; 2018.

[28] Macpherson H, Pipingas A, Pase MP. Multivitamin-multimineral supplementation and mortality: a meta-analysis of randomized controlled trials. Am J Clin Nutr. 2013;97:437–44. 23255568 10.3945/ajcn.112.049304

[29] Panel NS-o-tS. National Institutes of Health State-of-the-Science Conference Statement: multivitamin/mineral supplements and chronic disease prevention. Am J Clin Nutr. 2007;85:257S–64S. 17209206 10.1093/ajcn/85.1.257S

[30] Scholey A, Bauer I, Neale C, Savage K, Camfield D, White D, et al. Acute effects of different multivitamin mineral preparations with and without guaraná on mood, cognitive performance and functional brain activation. Nutrients. 2013;5:3589–604. 24067387 10.3390/nu5093589 PMC3798923

[31] Benton D, Haller J, Fordy J. Vitamin supplementation for 1 year improves mood. Neuropsychobiology. 1995;32:98–105. 7477807 10.1159/000119220

[32] Long S-J, Benton D. Effects of vitamin and mineral supplementation on stress, mild psychiatric symptoms, and mood in nonclinical samples: a meta-analysis. Psychosom Med. 2013;75:144–53. 23362497 10.1097/PSY.0b013e31827d5fbd

[33] Albert PR. Why is depression more prevalent in women?. J Psychiatry Neurosci. 2015;40:219–21. 26107348 10.1503/jpn.150205 PMC4478054

[34] Limm H, Angerer P, Heinmueller M, Marten-Mittag B, Nater UM, Guendel H. Self-perceived stress reactivity is an indicator of psychosocial impairment at the workplace. BMC Public Health. 2010;10:1–10. 20470413 10.1186/1471-2458-10-252 PMC2881886

[35] Schlotz W, Yim IS, Zoccola PM, Jansen L, Schulz P. The Perceived Stress Reactivity Scale: measurement invariance, stability, and validity in three countries. Psychol Assess. 2011;23:80. 21280954 10.1037/a0021148

[36] Turner AI, Smyth N, Hall SJ, Torres SJ, Hussein M, Jayasinghe SU, et al. Psychological stress reactivity and future health and disease outcomes: A systematic review of prospective evidence. Psychoneuroendocrinology. 2020;114:104599. 32045797 10.1016/j.psyneuen.2020.104599

[37] Kelly MM, Tyrka AR, Anderson GM, Price LH, Carpenter LL. Sex differences in emotional and physiological responses to the Trier Social Stress Test. J Behav Ther Exp psychiatry. 2008;39:87–98. 17466262 10.1016/j.jbtep.2007.02.003 PMC4467692

[38] Campagne DM. Stress and perceived social isolation (loneliness). Arch Gerontol Geriatr. 2019;82:192–9. 30825769 10.1016/j.archger.2019.02.007

[39] Victor CR, Yang K. The prevalence of loneliness among adults: a case study of the United Kingdom. J Psychol. 2012;146:85–104. 22303614 10.1080/00223980.2011.613875

[40] Eskelinen K, Hartikainen S, Nykänen I. Is loneliness associated with malnutrition in older people? Int J Gerontol. 2016;10:43–5.

[41] Pek K, Chew J, Lim JP, Yew S, Tan CN, Yeo A, et al. Social frailty is independently associated with mood, nutrition, physical performance, and physical activity: Insights from a theory-guided approach. Int J Environ Res Public Health. 2020;17:4239. 32545853 10.3390/ijerph17124239 PMC7345462

[42] Lapane KL, Lim E, McPhillips E, Barooah A, Yuan Y, Dube CE. Health effects of loneliness and social isolation in older adults living in congregate long term care settings: A systematic review of quantitative and qualitative evidence. Arch Gerontol Geriatr. 2022;102:104728. 35597183 10.1016/j.archger.2022.104728 PMC12102736

[43] Conolly A, Davies B. Health survey for England 2017: adult and child overweight and obesity. NHS Digital. 2018;1:1–30.

[44] Osborne B, Cooper V. Health Survey for England 2017 Adult health related behaviours. NHS Digital: Leeds, UK. 2018.

[45] Thorpe MG, Milte CM, Crawford D, McNaughton SA. Education and lifestyle predict change in dietary patterns and diet quality of adults 55 years and over. Nutr J. 2019;18:1–13. 31699092 10.1186/s12937-019-0495-6 PMC6839215

[46] Young LM, Gauci S, Scholey A, White DJ, Pipingas A. Self-selection bias: an essential design consideration for nutrition trials in healthy populations. Front Nutr. 2020;7:587983. 33240921 10.3389/fnut.2020.587983 PMC7683507

[47] Hicks S, Tinkler L, Allin P. Measuring subjective well-being and its potential role in policy: Perspectives from the UK office for national statistics. Soc Indic Res. 2013;114:73–86.

[48] Cohen S, Kamarck T, Mermelstein R. A global measure of perceived stress. J Health Social Behavior. 1983;24:385–96. 6668417

[49] Zigmond AS, Snaith RP. The hospital anxiety and depression scale. Acta Psychiatr Scandinavica. 1983;67:361–70. 10.1111/j.1600-0447.1983.tb09716.x 6880820

[50] McNair DM, Lorr M, Droppleman LF. Manual profile of mood states. 1971.

[51] Crawford J, Smith G, Maylor E, Della Sala S, Logie R. The Prospective and Retrospective Memory Questionnaire (PRMQ): Normative data and latent structure in a large non-clinical sample. Memory. 2003;11:261–75. 12908675 10.1080/09658210244000027

[52] Cohen S, Hoberman HM. Positive events and social supports as buffers of life change stress 1. J Appl Soc Psychol. 1983;13:99–125.

[53] Stewart AL, Hays RD, Ware JE. The MOS short-form general health survey: reliability and validity in a patient population. Med Care. 1988;26:724–35. 3393032 10.1097/00005650-198807000-00007

[54] Buysse DJ, Reynolds III, CF, Monk TH, Berman SR, Kupfer DJ. The Pittsburgh Sleep Quality Index: a new instrument for psychiatric practice and research. Psychiatry Res. 1989;28:193–213. 2748771 10.1016/0165-1781(89)90047-4

[55] Dipietro L, Caspersen CJ, Ostfeld AM, Nadel ER. A survey for assessing physical activity among older adults. Med Sci Sports Exerc. 1993;25:628–42. 8492692

[56] Yardley L, Beyer N, Hauer K, Kempen G, Piot-Ziegler C, Todd C. Development and initial validation of the Falls Efficacy Scale-International (FES-I). Age Ageing. 2005;34:614–9. 16267188 10.1093/ageing/afi196

[57] Antonucci TC, Akiyama H. Social networks in adult life and a preliminary examination of the convoy model. J Gerontol. 1987;42:519–27. 3624811 10.1093/geronj/42.5.519

[58] Lubben JE. Assessing social networks among elderly populations. Fam Community Health. 1988;11:42–52.

[59] De Jong-Gierveld J, Kamphuls F. The development of a Rasch-type loneliness scale. Appl Psychol Meas. 1985;9:289–99.

[60] Bingham SA, Welch AA, McTaggart A, Mulligan AA, Runswick SA, Luben R, et al. Nutritional methods in the European prospective investigation of cancer in Norfolk. Public Health Nutr. 2001;4:847–58. 11415493 10.1079/phn2000102

