# Supplementary material for: Improvements to mood, stress and loneliness following 12-week multivitamin supplementation in older adults: a randomised, placebo-controlled, trial
Source: Eur J Clin Nutr. 2024 Oct 3;79(2):126–35. doi: 10.1038/s41430-024-01517-6 (PMC11810787; doi:10.1038/s41430-024-01517-6)
Supplement: Supplementary file 1 — Table S1 [file 41430_2024_1517_MOESM1_ESM.pdf]

1. **Health conditions present at enrolment**

Table S1. Frequency of medical conditions in those enrolled in the main intervention.

Medical Condition	Number of Participants
Acid Reflux	46
Angina	2
Anxiety	2
Arthritis	20
Asthma	9
Breast Cancer (in remission)	4
COPD	6
Depression	22
Diabetes	24
Diverticular Disease	2
Emphysema	1
Enlarged Prostate	26
Glaucoma	4
Gout	10
Heart Attack	4
Heart Bypass	2
Heart Failure	3
High Blood Pressure	95
High Cholesterol	97
Hypothyroidism	17
MS	1
Nerve Damage	3
Osteoporosis	7
Overactive Bladder	3
Prostate cancer (in remission)	4
Sjogren's Syndrome	2
Stroke	3
